# Bone Morphogenetic Protein Signaling Protects against Cerulein-Induced Pancreatic Fibrosis

**DOI:** 10.1371/journal.pone.0089114

**Published:** 2014-02-21

**Authors:** Xuxia Gao, Yanna Cao, Dustin A. Staloch, Michael A. Gonzales, Judith F. Aronson, Celia Chao, Mark R. Hellmich, Tien C. Ko

**Affiliations:** 1 Department of Surgery, The University of Texas Health Science Center-Houston, Houston, Texas, United States of America; 2 Department of Pathology, University of Texas Medical Branch, Galveston, Texas, United States of America; 3 Department of Surgery, University of Texas Medical Branch, Galveston, Texas, United States of America; UAE University, Faculty of Medicine & Health Sciences, United Arab Emirates

## Abstract

Bone morphogenetic proteins (BMPs) have an anti-fibrogenic function in the kidney, lung, and liver. However, their role in chronic pancreatitis (CP) is unknown. The aim of this study was to define the anti-fibrogenic role of BMP signaling in the pancreas *in vivo* under CP induction. Mice with a deletion of BMP type II receptor (BMPR2^+/−^) were used in this study in comparison with wild-type mice. CP was induced by repetitive cerulein injection intraperitoneally for 4 weeks, and the severity of CP was evaluated. Pancreatic stellate cells (PSCs) were isolated from the mice and treated with BMP2 and TGF-β *in vitro*, and extracellular matrix protein (ECM) production was measured. Smad and *mitogen-activated protein kinase* (MAPK) signaling was also evaluated. BMPR2^+/−^ mice revealed a greater pancreatic fibrosis, PSC activation and leukocyte infiltration after CP induction compared to wild-type mice (*P*<0.05). Under CP induction, phospho (p)Smad1/5/8 was elevated in wild-type mice and this effect was abolished in BMPR2^+/−^ mice; pSmad2 and pp38^MAPK^ were further enhanced in BMPR2^+/−^ mice compared to wild-type mice (*P*<0.05). *In vitro*, BMP2 inhibited TGF-β-induced ECM protein fibronectin production in wild-type PSCs; this effect was abolished in BMPR2^+/−^ PSCs (*P*<0.05). In BMPR2^+/−^ PSCs, pSmad1/5/8 level was barely detectable upon BMP2 stimulation, while pSmad2 level was further enhanced by TGF-β stimulation, compared to wild-type PSCs (*P*<0.05). BMPR2/Smad1/5/8 signaling plays a protective role against cerulein-induced pancreatic fibrosis by inhibiting Smad2 and p38^MAPK^ signaling pathways.

## Introduction

Chronic pancreatitis (CP) is a progressive inflammatory disorder. The main characteristics of CP are acinar injury, leukocyte infiltration and pancreatic fibrosis, in which the destroyed pancreatic secretory parenchyma is replaced by fibrotic tissue, eventually resulting in malnutrition and diabetes [1]. Despite decades of research, no specific therapy is available; the treatment of CP remains empirical [2]. Therefore, better understanding of the mechanisms underlying CP pathophysiology is desired for development of specific and effective therapies.

Transforming growth factor (TGF)-β is a multifunctional protein with a broad spectrum of biological functions in cell growth, differentiation, and extra cellular matrix (ECM) production [3]. TGF-β plays a promoting role in fibrosis development in the kidney, lung, liver, and pancreas [4]–[7]. During CP, TGF-β is secreted by inflammatory cells and injured acinar cells. It activates quiescent pancreatic stellate cells (PSCs) into myofibroblast-like cells. These activated PSCs secrete TGF-β and produce excessive ECM proteins, leading to pancreatic fibrosis [8]–[10]. This pro-fibrogenic role of TGF-β is mediated by Smad2/3-dependent and Smad-independent pathway [11], [12]. Systemic blockade of TGF-β can cause detrimental problems due to the broad spectrum of biological functions of TGF-β in multiple organs. Therefore, targeting the pathways that can specifically modify or antagonize TGF-β signaling for pancreatic fibrosis development would be a more rational approach.

Bone morphogenetic protein (BMP) 2, a member of the TGF-β superfamily, has been implicated in the development of the skeleton, kidney and pancreas [13]–[15]. It functions by binding to the type II receptor (BMPR2) and recruiting a type I receptor (BMPR1a, BMPR1b or ACVR1), then phosphorylating intracellular substrates, i.e., Smad1, Smad5, and Smad8, which form complex with Smad4, translocate into the nucleus and regulate the transcription of various targets [16]. BMP2 has an anti-fibrogenic function in multiple organs. For instance, it antagonizes TGF-β-induced renal fibrogenic signals in renal fibroblasts and is effective in the treatment of rat renal fibrosis induced by unilateral urethral obstruction [17], [18]. BMP2 prevents differentiation and cell migration of lung fibroblasts [19]. BMP2 also attenuates pressure overload-induced cardiac fibrosis [20]. However, whether BMP2 has an anti-fibrogenic function in the pancreas is largely unknown.

Our recent study demonstrates that BMP2 inhibits TGF-β-induced PSC activation and ECM formation through Smad1 signaling pathway *in vitro* [21], suggesting an anti-fibrogenic role of BMP signaling in the pancreas. To investigate the role of BMP signaling pathway in CP, BMPR2/Smad1/5/8 signaling was blocked in the mice with a genetic deletion of BMPR2 (BMPR2^+/−^). These mice developed more severe pancreatic fibrosis under CP induction in comparison with wild-type mice, indicating a protective role of BMPR2/Smad1/5/8 signaling in pancreatic fibrosis. To our knowledge, this is the first demonstration of the anti-fibrogenic role of BMPR2/Smad1/5/8 signaling in the experimental CP model.

## Materials and Methods

### Reagents and Antibodies

Cerulein, the decapeptide analog of the potent pancreatic secretagogue cholecystokinin, was purchased from Bachem Americas, Inc. (Torrance, CA, USA). Human recombinant TGF-β1 and BMP2 were purchased from R&D Systems, Inc. (Minneapolis, MN, USA) and were diluted in a vehicle solution (0.1% BSA, 4 mM HCl). Antibodies against BMPR2, TGF-β1, α-smooth muscle actin (α-SMA) and CD45 were purchased from Abcam (Cambridge, MA, USA), phospho- and total- Smad2, Smad3, Smad1/5/8 and Smad1 from Cell Signaling Technology, Inc. (Billerica, MA, USA), phospho- and total-p38^MAPK^ from Invitrogen Corporation (Carlsbad, CA, USA), fibronectin (FN) and collagen Ia (ColIa) from Santa Cruz Biotechnology Inc. (Dallas, TX, USA), GAPDH from Sigma-Aldrich Corporate (St. Louis, MO, USA). Horseradish peroxidase (HRP) conjugated secondary antibodies were purchased from Bio-Rad Laboratories (Hercules, CA, USA).

### Animals

Animal experiments were performed according to the protocols approved by the Animal Welfare Committee of The University of Texas Health Science Center at Houston. Male BMPR2^+/−^ mice were kindly provided by Dr. H. Beppu (University of Toyama, Japan). These mice were crossed with female C57BL/6 mice (Charles River, Wilmington, MA, USA). Both males and females (8–10 weeks old) were used in the experiments. The animals were housed in a climate-controlled room with an ambient temperature of 23°C and a 12∶12-hour light-dark cycle. Animals were fed standard laboratory chow, given water ad libitum.

### *In vivo* Model of Chronic Pancreatitis

CP was induced in the mice by repetitive injections of cerulein [21]. Briefly, the mice were subjected to cerulein injection intraperitoneally (50 µg/kg, 5 hourly injections/day, 3 days/week) for 4 weeks. Normal saline was given at the same volume and frequency in control mice. The mice were euthanized at day 4 after completion of cerulein or normal saline injections. The pancreas was harvested for histological analysis and snap frozen for protein and RNA preparation.

### Morphological Examination

Pancreatic tissue samples were fixed in 10% formalin and subsequently embedded in paraffin. Five µm thick sections were prepared for hematoxylin-eosin (H&E) staining (Vector Laboratories, Inc., Burlingame, CA) and immunohistochemistry analysis.

### Quantitative Analysis of Pancreatic Fibrosis

Sirius red staining was performed on pancreas paraffin sections for the quantification of total intra-pancreatic collagen deposition [22]. Ten to fifteen images were taken from non-overlapping fields of each section. Sirius red-stained area was quantified using NIS-Elements Br 3.0 imaging analysis software and expressed as percentage of the total area measured.

### Immunohistochemistry Analysis

The pancreatic paraffin sections were prepared for immunohistochemistry analysis of α-SMA and CD45 with ABC kit and DAB kit (Vector Laboratories, Inc. Burlingame, CA, USA) according to the manufacturer’s instructions as described previously [21]. Briefly, following deparaffinization and hydration, antigen retrieval, blockage of endogenous peroxidase activity and nonspecific protein binding sites, the sections were then incubated with rabbit antibodies against α-SMA and CD45 overnight at 4°C. The sections were subsequently incubated with a biotinylated anti-rabbit antibody for one hour at room temperature, and then with ABC reagents for 30 minutes at room temperature. Finally the sections were stained using DAB kit followed by hematoxylin nuclei counterstaining and dehydrated, mounted with a permanent mounting solution (Vector Laboratories, Inc., Burlingame, CA, USA).

### Immunofluorescence

The frozen pancreatic tissue sections were dried for 60 minutes at room temperature, and then fixed with methanol at −20°C for 20 minutes. The PSCs were fixed with 4% paraformaldehyde for 15 minutes. Immunofluorescence was performed using TGF-β1, BMPR2 and FN antibodies as described before [21].

### Isolation and Culture of Mouse PSCs

Mouse primary PSCs were isolated from the pancreas of BMPR2^+/−^ and wild-type mice by an outgrowth method [23]. The cells were cultured in Dulbecco’s modified essential medium (DMEM, Mediatech Inc., Manassas, VA, USA) supplemented with 10% fetal bovine serum and 1% penicillin/streptomycin (Life Technologies-Invitrogen, Grand Island, NY, USA) at 37°C in a humidified incubator (containing 95% of O_2_ and 5% of CO_2_). Passages 1 to 3 were used.

### Western Blotting

Protein lysates were prepared from mouse pancreatic tissue samples or cells in 1X lysis buffer (Cell Signaling Technology, Inc., Billerica, MA, USA). Protein concentrations were measured using a protein assay dye (Bio-Rad Laboratories, Hercules, CA, USA). Western blotting analysis was performed as described previously [24].

### Quantitative Polymerase Chain Reaction (qPCR)

Total RNA was extracted from pancreatic tissue samples of the mice, and reversely transcribed to cDNA using RETROscript kit (Life Technology Co., Grand Island, NY, USA). qPCR was performed using Taqman gene expression master mix and specific gene probe sets as previously described [24]. The probe sets of mouse BMP2 (Mm013401798_m1), BMP4 (Mm00432087_m1), BMP7 (Mm00432102_m1), BMPR1a (Mm00477650_m1), BMPR2 (Mm00432134_m1), TGF-β1 (Mm01178820_m1), and 18s (Hs99999901_s1) (Life Technology Co., Grand Island, NY, USA) were used in the study. The signals acquired from BMPs, BMPRs, and TGF-β1 were normalized to the signals acquired from 18s, and expressed as fold of control.

### Statistical Analysis

Data were expressed as means ± SEM. *In vitro* experiments were repeated 2–3 times and similar results were obtained. Differences between two groups were analyzed using the Student’s *t*-test. Differences among multiple groups were analyzed using ANOVA with Tukey-Kramer multiple comparison test. *P*<0.05 is considered significant.

## Results

### The Levels of BMP Signaling Molecules Increased in the Pancreas Under CP Induction

**Figure 1:**
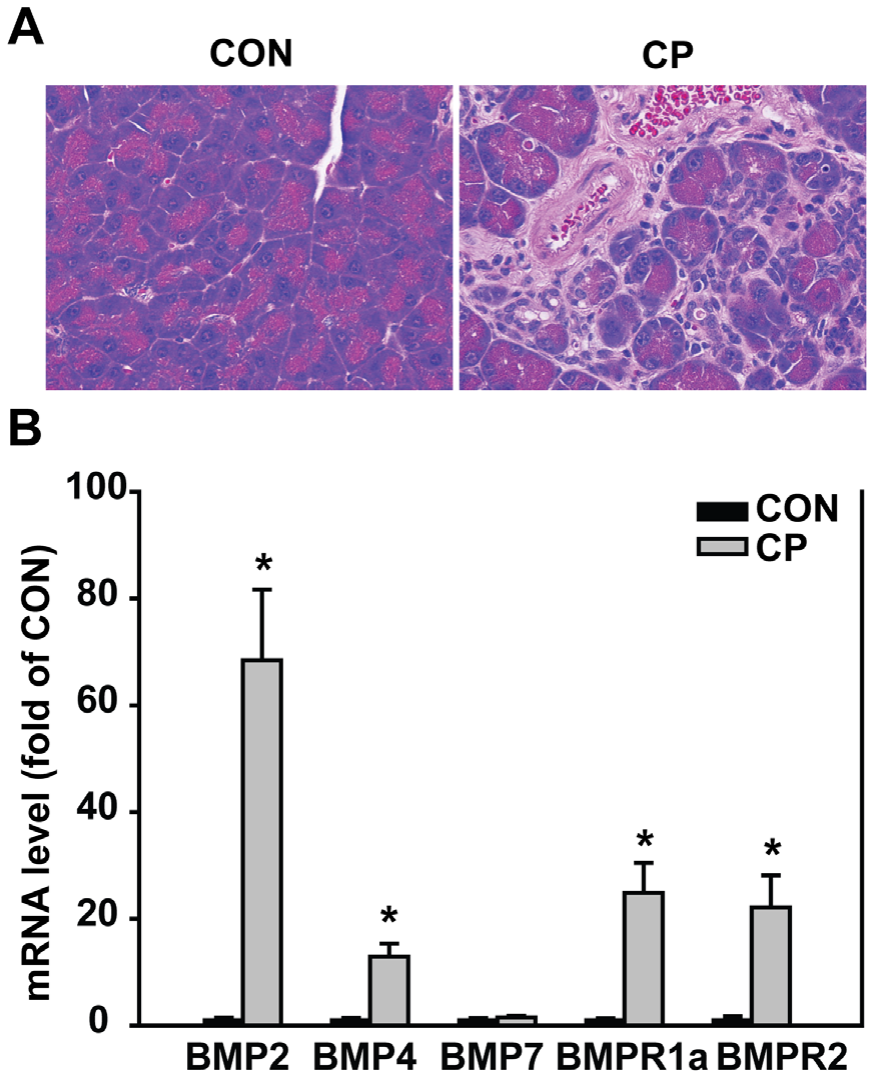
Expression of BMP signaling molecules increased in the pancreas under CP induction. (A) Representative images of H&E staining. Original magnification×400. (B) mRNA expression of BMP2, BMP4, BMP7, BMPR1a and BMPR2. The mRNA levels were normalized against 18s and quantified as fold of control (CON). *P<0.05 compared with CON. CON mice: n = 4, CP mice: n = 7.

In our previous study using Swiss Webster mice, we observed moderate fibrosis, increased BMP2 and pSmad1 levels in the pancreas of the mice receiving 4 weeks of cerulein injection [21]. Similarly in this study, C57BL/6 mice receiving 4 weeks of cerulein injection developed morphologic signs of CP with pancreatic fibrosis and leukocyte infiltration demonstrated by H&E staining (Figure 1A). Furthermore, the level of pancreatic BMP ligands BMP2 dramatically increased by 68 folds, BMP4 also increased by 13 folds, while BMP7 was unaltered, in CP mice compared to control mice. The levels of BMP receptors BMPR1a and BMPR2 increased by 25 folds and 22 folds, respectively, in CP mice compared to control mice (Figure 1B). These indicate an activated BMP signaling in cerulein-induced CP at 4 weeks.

### Characterization of BMPR2^+/−^ Mice

BMPR2 is a type II serine/threonine kinase receptor, which transduces signals from BMPs by forming heteromeric complexes with type I receptors. In our current study, BMPR2 and BMPR1a mRNA levels increased in the mouse pancreas with CP induction, along with BMP2 and BMP4, but not BMP7, implicating the potential roles of BMPRs in CP. To investigate the role of BMP signaling in the pancreas, we used a mouse line with a genetic deletion of BMPR2 in this study. BMPR2 deletion in homozygous mice (BMPR2^−/−^) results in embryonic lethality [16], heterozygous BMPR2^+/−^ mice are phenotypically normal and can develop pulmonary hypertension upon inflammatory insults [25], [26]. Therefore, the heterozygous BMPR2^+/−^ mice were used under CP induction in comparison with their littermate wild-type mice. Through immunofluorescence staining, we found that the BMPR2 protein level in the pancreas of BMPR2^+/−^ mice was 22% of wild-type mice. The adult BMPR2^+/−^ mice (8–10 weeks old) appeared healthy and their body weight was similar as the wild-type littermates. BMPR2^+/−^ mice showed normal pancreas morphology and normal blood glucose and insulin levels as wild-type mice (data not shown). These indicate that the BMPR2 heterozygous deficiency does not affect the pancreas development and the pancreatic endocrine and exocrine functions in adult mice.

### BMPR2 Deficiency Enhanced Pancreatic Fibrosis and PSC Activation Under CP Induction

**Figure 2:**
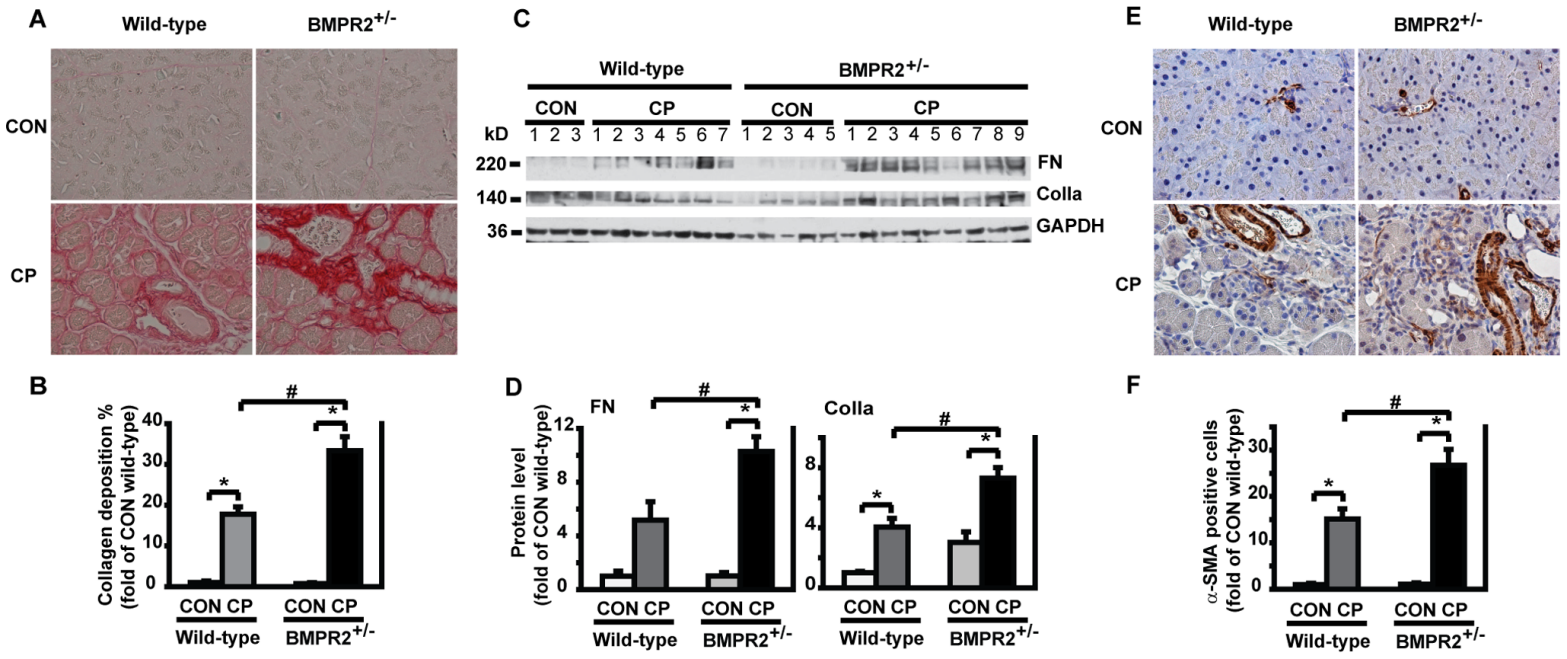
BMPR2 deficiency enhanced pancreatic fibrosis and PSC activation after CP induction. (A) Representative images of sirius red staining for collagen deposition in the pancreas. (B) Quantification of collagen deposition. CON wild-type: n = 4, CON BMPR2^+/−^: n = 5, CP wild-type: n = 7, CP BMPR2^+/−^: n = 9. Original magnification×400. (C) ColIa and FN protein levels in the pancreas by Western blotting. (D) Quantification of ColIa and FN. The protein levels were normalized against GAPDH and quantified as fold of CON wild-type. CON wild-type: n = 4, CON BMPR2^+/−^: n = 5, CP wild-type: n = 7, CP BMPR2^+/−^: n = 9. *P<0.05 compared with CON, ^#^ *P<*0.05 compared with CP wild-type. (E) Representative images of immunohistochemistry against α-SMA on paraffin-embedded pancreatic sections. (F) Quantification of α-SMA-positive cells in the periacinar areas. *n = *4 mice/group. Original magnification×400. *P<0.05 compared with CON, ^#^ *P<*0.05 compared with CP wild-type.

To investigate the role of BMPR2 on pancreatic fibrosis, BMPR2^+/−^ mice and wild-type mice were given cerulein for CP induction as described in Materials and Methods S1. Using sirius red staining in assessment of collagen deposition, we found that CP BMPR2^+/−^ mice exhibited more severe pancreatic fibrosis than CP wild-type mice (Figure 2A, B). Western blotting showed that the levels of ECM components FN and ColIa increased significantly in CP BMPR2^+/−^ mice compared to CP wild-type mice (Figure 2C, D). After 4 weeks of CP induction, the activated PSCs with α-SMA staining localized in the fibrotic areas around acini and increased markedly by 24 folds in wild-type mice and by 42 folds in BMPR2^+/−^ mice. There were significantly more activated PSCs in CP BMPR2^+/−^ mice than in CP wild-type mice (Figure 2E, F). These demonstrate that BMPR2 deletion led to an enhanced pancreatic fibrosis under CP induction.

Progressive fibrosis and destruction of the gland can ultimately lead to endocrine dysfunction. To evaluate implications of BMPR2 deficiency on pancreatic function following 4 weeks of CP induction, fasting blood insulin levels were measured and intraperitoneal glucose tolerance testing (IPGTT) was conducted. Fasting insulin levels showed no difference between wild-type and BMPR2^+/−^ mice or between control and CP mice; furthermore, IPGTT showed similar, normal response patterns to glucose challenge in all groups (Figure S1A, B). These indicate that CP induction with cerulein alone for 4 weeks is not sufficient to cause evident endocrine dysfunction in either wild-type or BMPR2^+/−^ mice.

### BMPR2 Deficiency Increased Leukocyte Infiltration in the Pancreas after CP Induction

**Figure 3:**
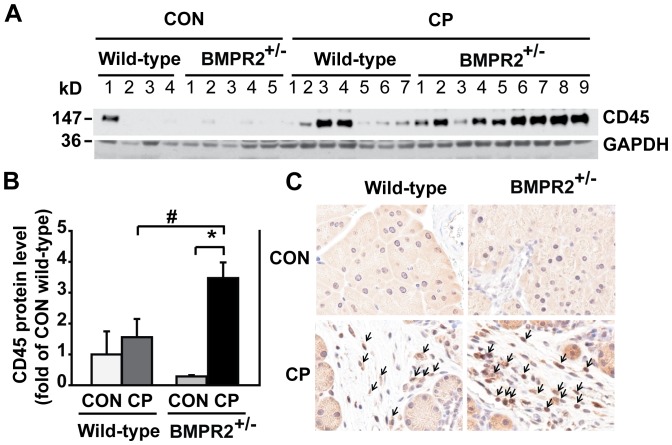
BMPR2 deficiency increased leukocyte infiltration in the pancreas after CP induction. (A) Western blotting of pancreatic tissue lysates with anti-CD45 antibody. (B) Quantification of CD45 Western blots. The protein levels were normalized against GAPDH and quantified as fold of CON wild-type. CON wild-type: n = 4, CON BMPR2^+/−^: n = 5, CP wild-type: n = 7, CP BMPR2^+/−^: n = 9. *P<0.05 compared with CON, ^#^ *P<*0.05 compared with CP wild-type. (C) Representative images of immunohistochemistry on paraffin-embedded pancreatic sections against CD45. Arrows point to CD45 positive cells. Original magnification×400.

To evaluate inflammatory infiltration, CD45, a marker of leukocytes, was measured by Western blotting and immunohistochemistry analysis. We found that CD45 protein expression increased significantly in CP BMPR2^+/−^ mice by Western blotting (Figure 3A, B). These results were confirmed by immunohistochemistry as shown in Figure 3C. In control wild-type and BMPR2^+/−^ mice, only a few leukocytes were spotted in the pancreas; while in CP BMPR2^+/−^ mice, a significant increase of leukocytes were observed compared to CP wild-type mice.

### BMPR2 Deficiency Enhanced pSmad2 Level under CP Induction

**Figure 4:**
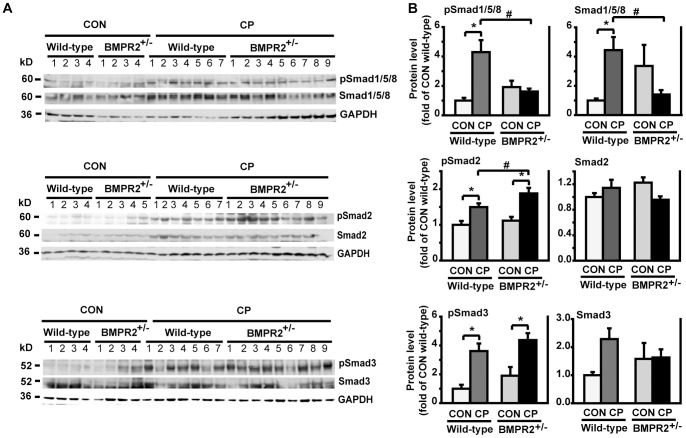
BMPR2 deficiency enhanced pSmad2 level after CP induction. (A) Western blotting of pancreatic tissue lysates with anti-pSmad1/5/8, -pSmad2, -pSmad3, total-Smad1/5/8, -Smad2 and -Smad3 antibodies. (B) Quantification of the Western blots. The protein levels were normalized against GAPDH and quantified as fold of CON wild-type. CON wild-type: n = 4, CON BMPR2^+/−^: n = 4–5, CP wild-type: n = 7, CP BMPR2^+/−^: n = 9. *P<0.05 compared with CON, ^#^ *P<*0.05 compared with CP wild-type.

Our previous data showed that pancreatic BMP2 and pSmad1 signaling increased at 4 weeks’ CP induction [21]. Similarly, in the current study, pSmad1/5/8 increased significantly in CP wild-type mice compared to control wild-type. However, this effect was not observed in CP BMPR2^+/−^ mice, indicating that BMPR2 heterozygous deficiency blocks pSmad1/5/8 signaling effectively. To investigate whether blocking BMP signaling affects TGF-β signaling, we evaluated pSmad2 and pSmad3 levels that have been reported to mediate the pro-fibrogenic function of TGF-β [12], [27]. Both pSmad2 and pSmad3 levels were elevated in wild-type and BMPR2^+/−^ mice after CP induction. However, with CP induction, only pSmad2 level was greater in BMPR2^+/−^ mice than in wild-type mice, while pSmad3 level was similar in BMPR2^+/−^ and wild-type mice (Figure 4). To investigate whether the increase of pSmad2 level in CP BMPR2^+/−^ was due to an increase of TGF-β1 level, we measured TGF-β1 on both mRNA and protein levels. TGF-β1 mRNA level increased significantly in CP mice compared to control mice, but there was no significant difference between wild-type and BMPR2^+/−^ mice under CP induction (Figure S2A). Similar patterns of TGF-β1 protein expression in the pancreas were found using immunofluorescence staining (Figure S2B). These indicate that BMP signaling does not inhibit TGF-β ligand production, and suggest that BMP signaling interacts with TGF-β signaling possibly at the TGF-β receptor levels, as reported in the renal fibrosis study [18]. In addition, inflammatory markers, TNF-α and IL-1β, in the pancreas were measured. Their mRNA levels were not elevated under induction of the chronic pancreatitis for 4 weeks (data not shown). The lack of change in the level of these acute inflammatory mediators is not unexpected since this is a chronic pancreatitis model induced by recurrent acute pancreatitis (RAP). Within the context of RAP, there are well documented increases in the levels of TNF-α and IL-1β during the period of repeated cerulein challenge. However, these increases are transient and the levels return to baseline when the treatment ends [28], [29].

### BMPR2 Deficiency Elevated p38^MAPK^ Signaling under CP Induction

**Figure 5:**
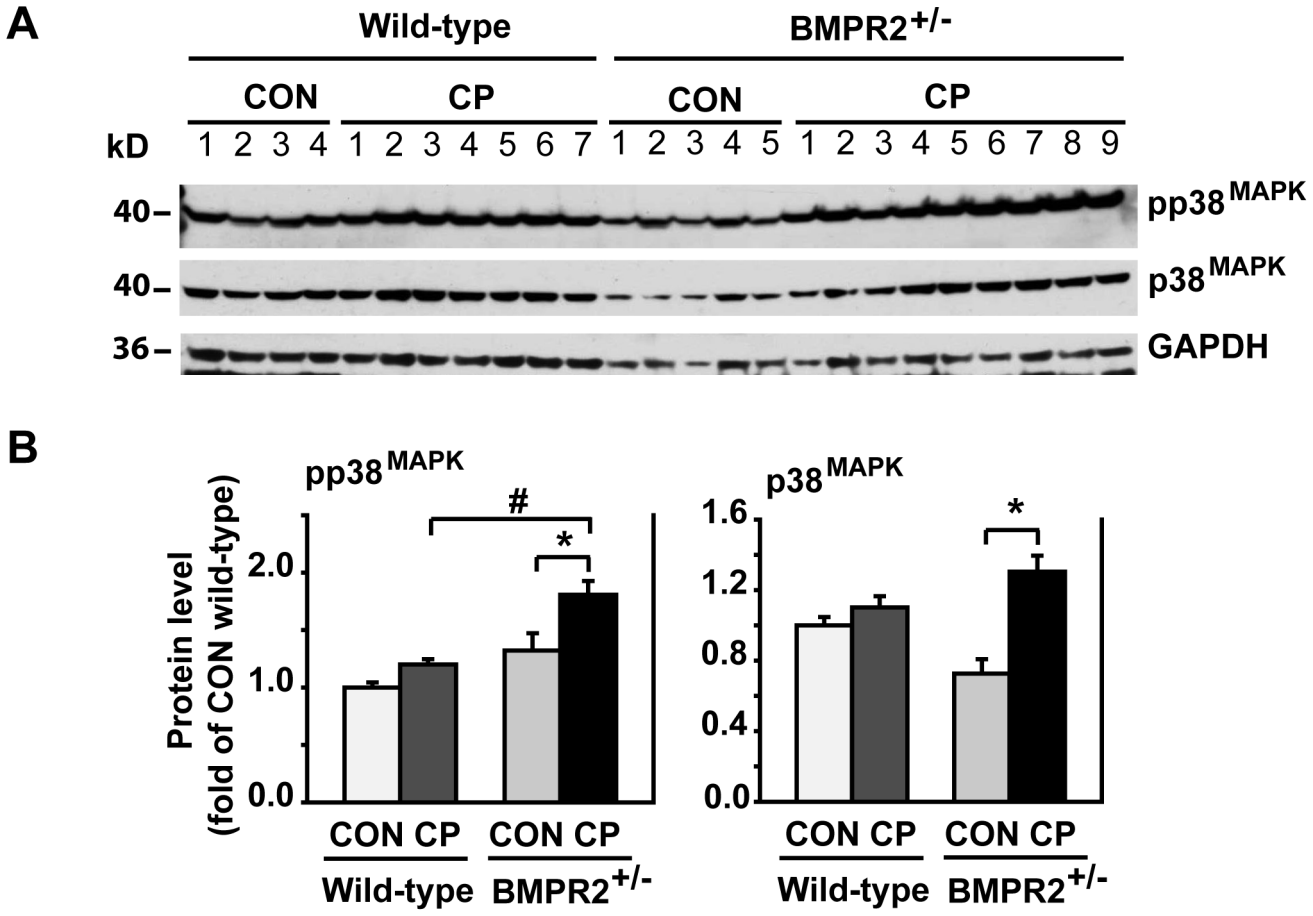
pp38^MAPK^ signaling increased in BMPR2^+/−^ mice after CP induction. (A) Western blotting of pancreatic tissue lysates from wild-type and BMPR2^+/−^ mice with anti-pp38^MAPK^ and total p38^MAPK^ antibodies. (B) Quantification of the Western blots. The protein levels were normalized against GAPDH and quantified as fold of CON wild-type. CON wild-type: n = 4, CON BMPR2^+/−^: n = 5, CP wild-type: n = 7, CP BMPR2^+/−^: n = 9. *P<0.05 compared with CON, ^#^ *P<*0.05 compared with CP wild-type.

p38^MAPK^ signaling pathway participates in the development of CP as well as Smad signaling pathway [30]. We further assessed pp38^MAPK^. Our data showed that pp38^MAPK^ was elevated in CP BMPR2^+/−^ mice but not in CP wild-type mice (Figure 5). These data suggest that the protective role of BMP/Smad1/5/8 signaling is likely through inhibiting TGF-β/Smad2 and/p38^MAPK^ signaling.

### BMP2/pSmad1/5/8 Signaling Inhibited TGF-β-induced Fibronectin in PSCs

**Figure 6:**
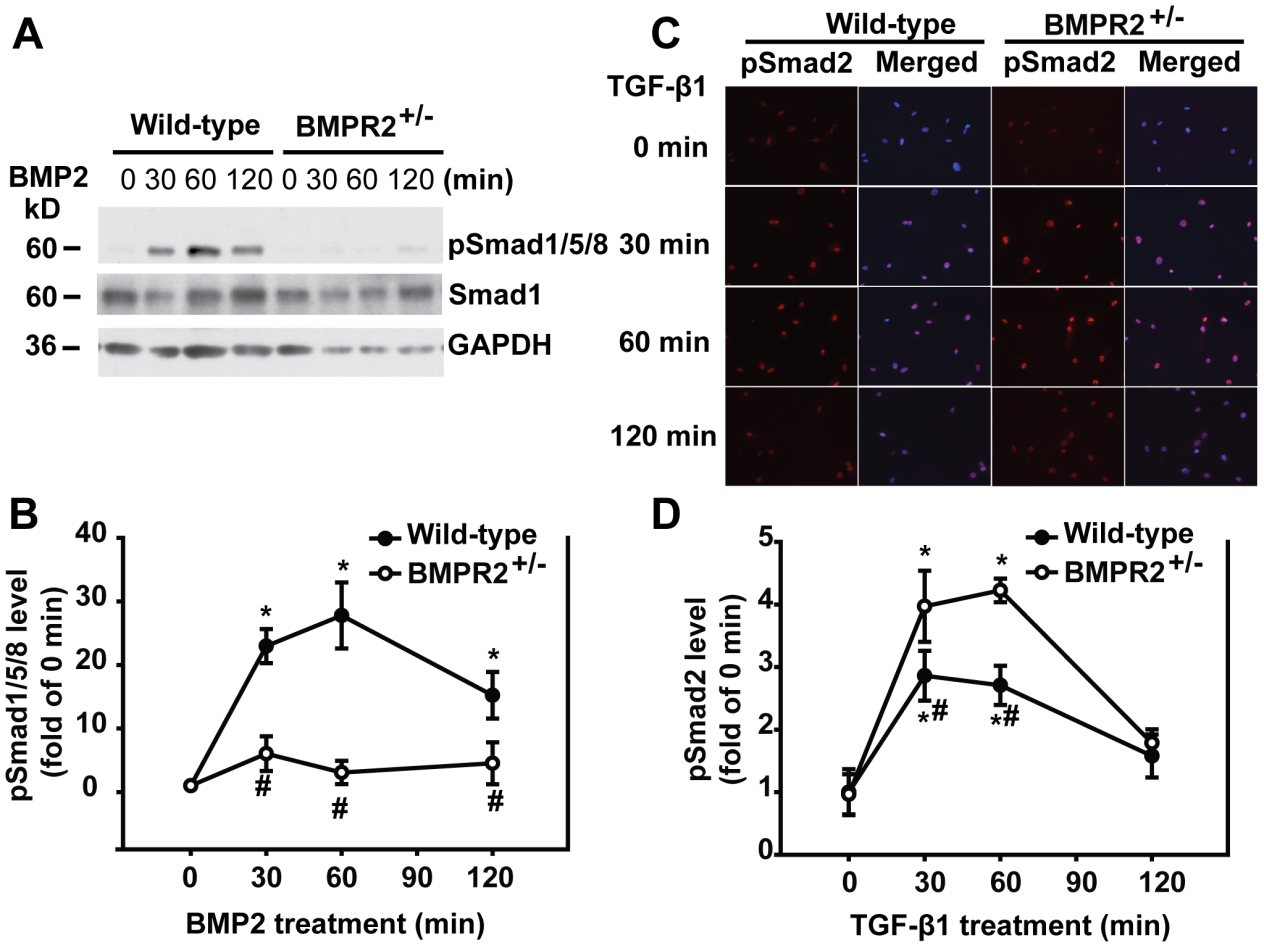
BMPR2 deficiency increased Smad2 phosphorylation in PSCs. (A) Wild-type and BMPR2^+/−^ PSCs were treated with 250 ng/ml of BMP2 for the indicated time points. The whole cell lysates were subjected to Western blotting with anti-pSmad1/5/8 and total Smad1 antibodies. (B) Quantification of the Western blots. The protein levels were normalized against GAPDH and quantified as fold of 0 min. *P<0.05 compared with 0 min, ^#^ *P<*0.05 compared with the same time points of wild-type. Wild-type: n = 3, BMPR2^+/−^: n = 3. (C) Wild-type and BMPR2^+/−^ PSCs were treated with 1 ng/ml of TGF-β1 for the indicated time points. Immunofluorescence was performed using antibody against pSmad2 (red). The cell nucleus was stained with DAPI (blue). The images of pSmad2 and pSmad2 merged with DAPI were presented. (D) Quantification of fluorescence intensity from 3 different fields per sample. *P<0.05 compared with 0 min, ^#^ *P<*0.05 compared with the same time points of wild-type PSCs. Original magnification×200.

**Figure 7:**
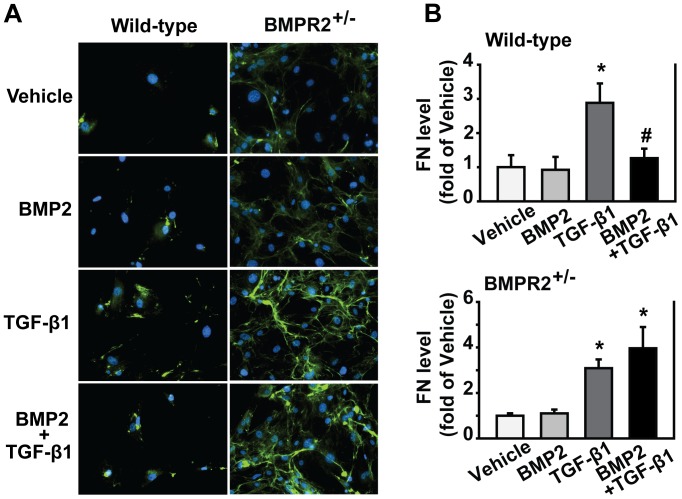
BMP2/pSmad1/5/8 signaling inhibited TGF-β-induced fibronectin in PSCs. (A) Wild-type and BMPR2^+/−^ PSCs were pretreated with 250 ng/ml of BMP2 for 30 min followed by 1 ng/ml of TGF-β1 for 48 h. Immunofluorescence was performed using antibody against FN (green), and the cell nucleus was stained with DAPI (blue). The images of FN merged with DAPI were presented. (B) Quantification of fluorescence intensity from 5–10 different fields per slide, *P<0.05 compared with vehicle group, ^#^ *P<*0.05 compared with TGF-β1 group. Original magnification×200.

To define the anti-fibrogenic role of BMP signaling *in vitro*, we isolated PSCs from BMPR2^+/−^ and wild-type mice. PSCs were treated with either BMP2 or TGF-β1 for 30, 60 or 120 minutes. pSmad1/5/8 levels were evaluated by Western blotting and pSmad2 levels were accessed by immunofluorescence. In response to BMP2 stimulation, pSmad1/5/8 levels were elevated beginning at 30 minutes and lasting up to 120 minutes in the PSCs from wild-type mice but not in the PSCs from BMPR2^+/−^ mice (Figure 6A, B). Under TGF-β1 stimulation, pSmad2 levels were elevated in PSCs from both wild-type and BMPR2^+/−^ mice. However, pSmad2 levels were greater in the PSCs from BMPR2^+/−^ mice than that in the PSCs from wild-type mice (Figure 6C, D). The PSCs were also pretreated with BMP2 (250 ng/ml) for 30 minutes followed by TGF-β1 (1 ng/ml) for 48 hours for evaluation of ECM formation. Shown in Figure 7, BMP2 significantly inhibited TGF-β-induced FN in PSCs from wild-type mice, while the inhibitory effect was abolished in PSCs from BMPR2^+/−^ mice. These data indicate that BMP2/pSmad1/5/8 signaling protects against fibrosis through inhibiting TGF-β/Smad2 signaling.

## Discussion

BMP2 plays an anti-fibrogenic role in multiple organs [17], [19], [20]. However, the function of BMP2 in the development of pancreatic fibrosis remains unclear. We have demonstrated previously that BMP2 inhibits TGF-β-induced PSC activation and ECM formation *in vitro* [21]. In this study, we used BMPR2^+/−^ mice and demonstrated that BMPR2/Smad1/5/8 signaling plays an anti-fibrogenic role in the pancreas, which was probably through inhibiting TGF-β/Smad2 and/p38^MAPK^ signaling pathways.

BMPR2 is one of the three BMP type II receptors (BMPR2, ActRIIa and ActRIIb) [31]. Mutations of the BMPR2 gene have been implicated in the pathogenesis of pulmonary arterial hypertension (PAH) in human [25]. In mice, BMPR2 homozygous deficiency is embryonic lethal [32], while heterozygous mice (BMPR2^+/−^) are phenotypically normal [16]. These mice are susceptible to the secondary inflammatory insults such as 5-lipoxygenase and develop PAH [25]. Similarly, BMPR2^+/−^ mice in our study were morphologically healthy, presented normal pancreatic histology and function. A comparable basal pSmad1/5/8 level in the pancreas was observed in BMPR2^+/−^ mice as in wild-type control (Figure 4), indicating a functional pancreatic BMP signaling existing in the heterozygous mice, which may account for the normal development of the pancreas.

Under CP induction, the pancreatic pSmad1/5/8 level was elevated in wild-type mice, whereas this effect was not seen in BMPR2^+/−^ mice (Figure 4). Furthermore, more severe pancreatic fibrosis and activated PSCs were observed in BMPR2^+/−^ mice (Figure 2). *In vitro,* in response to BMP2 stimulation, pSmad1/5/8 level was elevated in PSCs isolated from wild-type mice, and this effect was abolished in PSCs from BMPR2^+/−^ mice (Figure 6). Subsequently, the PSCs from BMPR2^+/−^ mice lost BMP2′s inhibitory effect on TGF-β-induced ECM production (Figure 7). Together these *in vivo* and *in vitro* results establish the anti-fibrogenic role of BMPR2/Smad1/5/8 signaling in the pancreas *via* CP model.

While more severe fibrosis following CP induction was observed in BMPR2 deficient mice versus their wild-type counterparts, neither persistent endocrine dysfunction nor overt diabetes was observed in either group. These results are not unexpected. Endocrine dysfunction and overt diabetes are late, inconsistent complications manifesting in roughly half of human cases, reflecting decreased insulin release with loss of islet cell mass in extensive disease [33]. Pancreatic diabetes mouse models have been developed with cerulein-induced pancreatitis. In these models, longer induction periods (16 week-cerulein injections) than what we employed in the current study (4 week-cerulein injections) were used, and a second insult was necessary to observe endocrine dysfunction [34]. These indicate both dose- and time-dependence of the disease extent and the minor direct effect of cerulein on the pancreatic endocrine cells. These aspects of BMP’s complex role in glucose metabolism during pancreatitis deserve further investigation.

BMPs and TGF-β belong to a same superfamily and play important roles in the regulation of cellular functions. They share a similar ligand structure and a similar downstream signaling cascade. Therefore, they might coordinately modulate organ fibrosis. Yang, et al. [18] demonstrated that BMP2 antagonized the pro-fibrogenic role of TGF-β in renal fibrosis through suppressing TGF-β receptor I thereby inhibiting Smad2/3 phosphorylation. In current study, we found that TGF-β1 and its downstream signaling pSmad2 and pSmad3 in the pancreas increased significantly under CP induction. Deficiency of BMP signaling in BMPR2^+/−^ mice further enhanced pSmad2 and pp38^MAPK^ levels in the pancreas under CP induction, but not pancreatic pSmad3 and TGF-β1 ligand levels. *In vitro*, deficiency of BMP signaling in PSCs isolated from BMPR2^+/−^ mice also enhanced pSmad2 level in response to TGF-β1 stimulation. Both *in vivo* and *in vitro* data indicate an antagonizing interaction of BMP and TGF-β signaling in the regulation of pancreatic fibrosis.

Both inflammation and fibrosis are characteristic features of CP. In this study, we found that the BMPR2 deficiency exacerbated inflammation in the pancreas, evidenced by an increased CD45 expression in the infiltrates after CP induction in BMPR2^+/−^ mice. These indicate that BMP signaling also plays an important anti-inflammatory role in the pancreas under CP induction. Further investigation is deserved on cellular and molecular mechanisms involved.

In conclusion, our results from the CP mouse model *in vivo* and the isolated PSCs *in vitro* demonstrate that the deficiency of BMP signaling aggravated cerulein-induced pancreatic fibrosis and abolished BMP2′s inhibitory effect on ECM production induced by TGF-β. Furthermore, the deficiency of BMP signaling leads to an enhanced pSmad2 and pp38^MAPK^ signaling. Our results indicate that BMPR2/Smad1/5/8 signaling pathway plays an anti-fibrogenic role in the pancreatic fibrosis, which is probably through inhibiting TGF-β/pSmad2 and/pp38^MAPK^ signaling pathways.

Studies in mouse models have shown that inhibition of TGF-β signaling with a dominant-negative mutant of TGF-β receptor II led to amelioration of pancreatic fibrosis [35], and inhibition of p38^MAPK^ with the specific inhibitor attenuated renal fibrosis [36]. Therefore, to further delineate the BMP’s protective role, in future experimentation, we plan to recapitulate the BMP2’s protective effects in the animals by inhibiting Smad2 and p38^MAPK^.

## Supporting Information

Figure S1 **Endocrine function in wild-type and BMPR2^+/−^ mice under chronic pancreatitis induction.** (A) Fasting insulin levels were measured by ELISA from mouse plasma collected after four weeks of cerulein injections. (B) IPGTT was performed utilizing an AccuChek glucometer from the mouse plasma collected at indicated time points after glucose injection. CON wild-type: n = 3, CON BMPR2**^+/−^**: n = 5, CP wild-type: n = 5, CP BMPR2**^+^** ^/**−**^: n = 5.
(TIF)

Figure S2 **BMPR2 deficiency did not affect the elevated TGF-β1 levels after CP induction.** (A) Pancreatic TGF-β1 mRNA levels were measured by qPCR. The mRNA levels were normalized against 18s and quantified as fold of CON wild-type. CON wild-type: n = 4, CON BMPR2^+/−^: n = 5, CP wild-type: n = 7, CP BMPR2^+/−^: n = 9. *P<0.001 compared CP with CON. (B) Representative images of immunofluorescence on frozen pancreatic sections with anti-TGF-β1 antibody (red) and nuclei stained with DAPI (blue). The arrows point to TGF-β1 positive staining. Original magnification,×200.
(TIF)

Materials and Methods S1 **Enzyme-linked immunosorbent assay for insulin.** Following four weeks of cerulein injection, BMPR2+/− and wild-type mice were subjected to a six-hour fast with access to drinking water. Approximately 20 µl of blood was collected from the mouse tail vein by a heparinized hematocrit tube (Drummond Scientific Company, Broomal, PA, USA) prior to glucose tolerance testing. Blood was spun at 2500 g for 10 min at room temperature in hemotubes to separate plasma. Plasma insulin levels were measured by ELISA (Crystal Chem Inc., Downers Grove, IL, USA) according to the protocol supplied by the manufacturer. Intraperitoneal glucose tolerance testing (IPGTT). BMPR2+/− and wild-type mice underwent repetitive cerulein intraperitoneal injections. After four weeks of injections, mice were subjected to a six-hour fast with access to drinking water. Mice were weighed and blood was collected from the mouse tail vein, and glucose levels were measured using a glucometer (Accu-Chek, Roche, NJ). After a fasting glucose level was obtained, glucose (2,000 mg/kg) was injected intraperitoneally according to the weight of the individual mouse, and blood glucose levels were measured at 10, 30, 60, 90 and 120 min postinjection.
(DOC)
