# Supplementary figures and images for: Bone Morphogenetic Protein Signaling Protects against Cerulein-Induced Pancreatic Fibrosis
Source: PLoS One. 2014 Feb 21;9(2):e89114. doi: 10.1371/journal.pone.0089114 (PMC3931685; doi:10.1371/journal.pone.0089114)

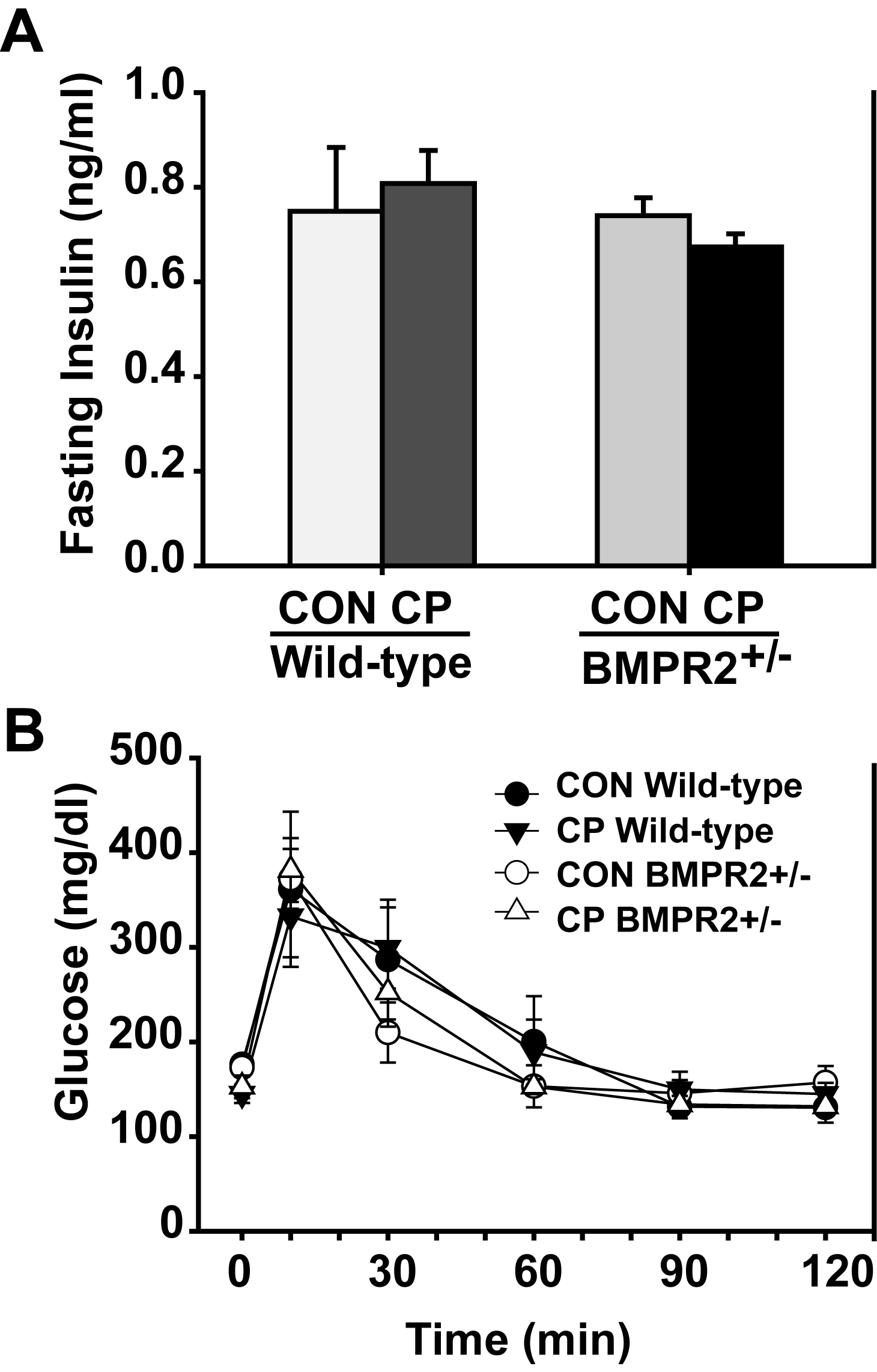

Supplement: Figure S1 — Endocrine function in wild-type and BMPR2+/− mice under chronic pancreatitis induction. (A) Fasting insulin levels were measured by ELISA from mouse plasma collected after four weeks of cerulein injections. (B) IPGTT was performed utilizing an AccuChek glucometer from the mouse plasma collected at indicated time points after glucose injection. CON wild-type: n = 3, CON BMPR2+/−: n = 5, CP wild-type: n = 5, CP BMPR2+ /−: n = 5. (TIF) [file pone.0089114.s001.tif]

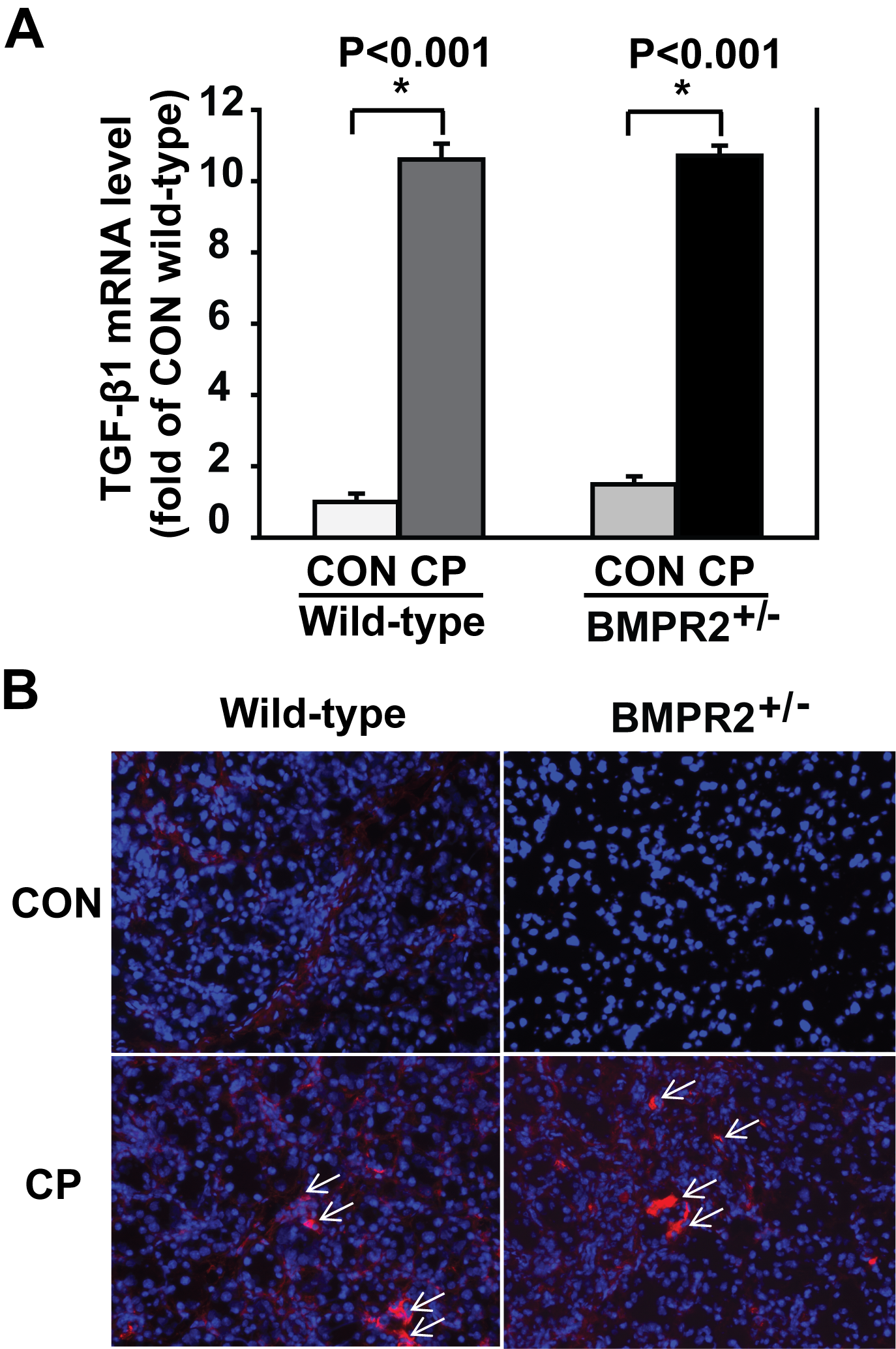

Supplement: Figure S2 — BMPR2 deficiency did not affect the elevated TGF-β1 levels after CP induction. (A) Pancreatic TGF-β1 mRNA levels were measured by qPCR. The mRNA levels were normalized against 18s and quantified as fold of CON wild-type. CON wild-type: n = 4, CON BMPR2+/−: n = 5, CP wild-type: n = 7, CP BMPR2+/−: n = 9. *P<0.001 compared CP with CON. (B) Representative images of immunofluorescence on frozen pancreatic sections with anti-TGF-β1 antibody (red) and nuclei stained with DAPI (blue). The arrows point to TGF-β1 positive staining. Original magnification,×200. (TIF) [file pone.0089114.s002.tif]
