# Supplementary material for: Bone Morphogenetic Protein Signaling Protects against Cerulein-Induced Pancreatic Fibrosis
Source: PLoS One. 2014 Feb 21;9(2):e89114. doi: 10.1371/journal.pone.0089114 (PMC3931685; doi:10.1371/journal.pone.0089114)
Supplement: Materials and Methods S1 — Enzyme-linked immunosorbent assay for insulin. Following four weeks of cerulein injection, BMPR2+/− and wild-type mice were subjected to a six-hour fast with access to drinking water. Approximately 20 µl of blood was collected from the mouse tail vein by a heparinized hematocrit tube (Drummond Scientific Company, Broomal, PA, USA) prior to glucose tolerance testing. Blood was spun at 2500 g for 10 min at room temperature in hemotubes to separate plasma. Plasma insulin levels were measured by ELISA (Crystal Chem Inc., Downers Grove, IL, USA) according to the protocol supplied by the manufacturer. Intraperitoneal glucose tolerance testing (IPGTT). BMPR2+/− and wild-type mice underwent repetitive cerulein intraperitoneal injections. After four weeks of injections, mice were subjected to a six-hour fast with access to drinking water. Mice were weighed and blood was collected from the mouse tail vein, and glucose levels were measured using a glucometer (Accu-Chek, Roche, NJ). After a fasting glucose level was obtained, glucose (2,000 mg/kg) was injected intraperitoneally according to the weight of the individual mouse, and blood glucose levels were measured at 10, 30, 60, 90 and 120 min postinjection. (DOC) [file pone.0089114.s003.doc]

**Supplemental Materials and Methods**

**Enzyme-linked immunosorbent assay for insulin**

Following four weeks of cerulein injection, BMPR2^+/-^ and wild-type mice were subjected to a six-hour fast with access to drinking water. Approximately 20 μl of blood was collected from the mouse tail vein by a heparinized hematocrit tube (Drummond Scientific Company, Broomal, PA, USA) prior to glucose tolerance testing. Blood was spun at 2500 g for 10 min at room temperature in hemotubes to separate plasma. Plasma insulin levels were measured by ELISA (Crystal Chem Inc., Downers Grove, IL, USA) according to the protocol supplied by the manufacturer.

**Intraperitoneal glucose tolerance testing (IPGTT)**

BMPR2^+/-^ and wild-type mice underwent repetitive cerulein intraperitoneal injections. After four weeks of injections, mice were subjected to a six-hour fast with access to drinking water. Mice were weighed and blood was collected from the mouse tail vein, and glucose levels were measured using a glucometer (Accu-Chek, Roche, NJ). After a fasting glucose level was obtained, glucose (2,000 mg/kg) was injected intraperitoneally according to the weight of the individual mouse, and blood glucose levels were measured at 10, 30, 60, 90 and 120 min postinjection.
